# Impact of midfoot and Hindfoot involvement on functional disability in Korean patients with rheumatoid arthritis

**DOI:** 10.1186/s12891-017-1726-7

**Published:** 2017-08-24

**Authors:** Hye-Jin Jeong, Il Woong Sohn, Dam Kim, Soo-Kyung Cho, Si-Bog Park, Il-Hoon Sung, Yoon-Kyoung Sung

**Affiliations:** 1 Department of Rheumatology, Keimyung University Dongsan Medical Center, Daegu, South Korea; 2 Department of Rheumatology, Hanyang University Hospital for Rheumatic Diseases, Seoul, South Korea; 3 Department of Rehabilitation Medicine, Hanyang University Hospital, Seoul, South Korea; 4 Department of Orthopedic Surgery, Hanyang University Hospital, Seoul, South Korea

**Keywords:** Functional disability, Health assessment questionnaire-disability index, Hindfoot, Midfoot, Rheumatoid arthritis

## Abstract

**Background:**

Foot involvement in rheumatoid arthritis (RA) patients has been reported to severely affect functional capacity and quality of life. We aimed to determine the impact of midfoot and hindfoot involvement on functional disability in Korean patients with RA.

**Methods:**

We evaluated the RA involvement and deformity of three regions of the foot (forefoot, midfoot and hindfoot) and ankle using conventional radiography in Korean patients with RA. We compared the clinical features between RA patients with and without foot or ankle involvement. Using multivariable logistic regression analyses, the impact of midfoot or hindfoot involvement on functional disability in RA patients was evaluated.

**Results:**

Overall, 120 patients with a median age of 48.0 [interquartile range (IQR), 37–56] years and median disease duration of 58.0 (IQR, 10–89) months were included. The prevalence of foot or ankle RA involvement was 74 (61.7%). The number of patients with forefoot, midfoot, hindfoot and ankle involvement was 32 (43.2%), 24 (32.4%), 46 (62.2%) and 4 (5.4%), respectively. Compared to patients without foot or ankle involvement those with such involvement had greater disease activity and functional disability, more of them were treated with biologic agents, and they had a lower health-related quality of life. After adjusting for potential confounders, hindfoot involvement was associated with a higher degree of functional disability. However, walking difficulty was more associated with midfoot involvement rather than with involvement in other regions.

**Conclusions:**

In Korean patients with RA, hindfoot involvement is associated with functional disability and midfoot involvement affects walking.

**Electronic supplementary material:**

The online version of this article (doi:10.1186/s12891-017-1726-7) contains supplementary material, which is available to authorized users.

## Background

Rheumatoid arthritis (RA) is a chronic inflammatory disease that affects multiple joints, especially the small joints of the hands and feet [1, 2]. Chronic joint inflammation results in synovial thickening, the production of excessive joint fluid, and damage to cartilage and bones, which can lead to permanent joint destruction and disability [1]. It is thought that in the early stages of the disease joint inflammation affects daily functioning mainly due to pain and swelling, while in later stages of the disease joint destruction and deformity increase functional impairment [3].

Foot involvement is a major cause of disability in RA [4], and can lead to a deterioration in daily activity and quality of life [5, 6]. Approximately 80–90% of patients with RA experience foot pain during the course of the disease [1, 5, 7, 8]. Yet, despite the large number of RA patients with foot complaints, these are frequently overlooked when caring for patients with multiple joint pains and deformities. For example, the disease activity score (DAS)-28 is the most widely used assessment tool of disease activity in RA, but the joints in the foot are not included in the 28-joint count [9]. Even patients in a remission state may suffer from foot pain [10, 11].

The foot is divided into three regions (the forefoot, midfoot and hindfoot). The forefoot is composed of the metatarsal bones and the phalanges connected by the metatarsophalangeal (MTP) and interphalangeal (IP) joints. The forefoot acts as a lever during the pressing phase of the gait. Forefoot deformity is characterized by hallux valgus and subluxation of the MTP joints [6]. The foot impairment caused by RA most commonly affects the forefoot, and symptoms begin in the MTP joints in most cases [5, 8, 12]. The midfoot joint, including naviculocuneiform joint and tarsometatarsal (TMT) joint, constitutes the longitudinal arch of the foot and allows the load to be transferred to the forefoot during gait [12, 13]. Additional functions provided by the midfoot are the ability to walk on uneven ground and to absorb shock [7]. The midfoot is not commonly affected on its own but is involved in 40–60% of patients with RA [6, 12]. The hindfoot joint, including the talonavicular, calcaneocuboid and talocalcaneal (also known as subtalar) joints, performs the side-to-side motion of the foot and provides stability at the initiation of the foot strike and is involved in 30–60% of RA cases [6, 12, 14]. It is also less frequently involved in the absence of midtarsal- and MTP joint disease. The ankle permits two types of movement, dorsiflexion and plantar flexion and contributes to lower limb stability [14]. The ankle joint is less commonly involved than other joints of the foot. RA involvement of the ankle joint is seen in approximately 9–26% of patients [4, 14, 15].

Numerous studies have reported an association between RA involvement or deformity of the forefoot in RA and functional disability [16–21]. In those cases, orthotic devices and surgical treatment are able to relieve pain and improve function [14]. However, there are a few studies on the effects of midfoot- and hindfoot arthritis in RA [15, 22, 23]. The purpose of the present study was to assess the prevalence of midfoot and hindfoot involvement and its impact on functional disability in Korean patients with RA.

## Methods

### Study population

A total of 120 RA patients were included in this study. It used basline data from published research on the impact of patient education on the satisfaction of Korean patients with RA in a rheumatology clinic of a tertiary referral hospital [24]. According to the protocol of this previous study, 120 patients were classified into 3 groups; 40 biologic disease-modifying antirheumatic drug (DMARD) users of any disease duration, 40 conventional DMARD users of less than 2 years of disease duration, and 40 conventional DMARD users with over 2 years of disease duration. All patients were over 18 years of age and fulfilled either the 2010 American College of Rheumatology/European League Against Rheumatism (ACR/EULAR) or the 1987 ACR classification criteria for RA. Patients with a diabetic foot ulcer, peripheral vascular disease, peripheral neuropathy, or foot fracture and previous surgery on the foot were excluded. All the patients voluntarily completed a consent form and the study was approved by Hanyang University Hospital Institutional Review Board (IRB No. 2014–08-023 & No. 2015–03-008).

### Data collection

Participants were interviewed face-to-face and also filled out self-completed questionnaires. Demographic data (age, sex) and clinical data (duration of disease, medications such as glucocorticoid, conventional and biological DMARD) were evaluated by face-to-face interview. We calculated the body mass index (BMI) (calculated as weight in kilograms divided by the square of height in meters) by measuring height and weight. Participants were examined the number of swollen/tender joints, patient’s global assessment on visual analog scale by interview. Laboratory data included C-reactive protein (CRP), ESR, and presence of rheumatoid factor (RF) and anti-cyclic citrullinated peptide antibody (ACPA) were collected. Disease activity was measured using the disease activity score 28 - erythrocyte sedimentation rate (DAS28-ESR) [9]. The Korean version of the EuroQol five-dimension questionnaire (EQ-5D), a tool for assessing quality of life, was evaluated by self-completed questionnaires [25].

### Radiologic measurement of RA involvement and deformity of the foot and ankle

All radiographic data were collected within one year of the enrollment date. Supine anteroposterior and weight-bearing lateral views of conventional radiographs of both feet were obtained for all subjects. Radiographs of both feet together were obtained in anteroposterior view, and radiographs of the individual feet were obtained separately in lateral view. These radiographs were independently assessed by two rheumatologists. In cases of discrepancies in the readings of the two examiners, the radiograph was inspected again and discussed until a consensus was reached. We evaluated RA involvement in three parts of the foot (forefoot, midfoot and hindfoot) and the ankle joint; the forefoot joint included the first to fifth MTP joints and the first IP joint; the midfoot joint included the first to fifth TMT joints (also known as the Lisfranc joint) and the naviculocuneiform joint; the hindfoot joint consisted of the talonavicular, calcaneocuboid and talocalcaneal joint; the ankle joint included tibiotalar joint. RA involvement was defined as the presence of erosion on least one side or a unilateral or bilateral joint space-narrowing. For example, the presence of erosion or joint space narrowing in either the unilateral or bilateral first-to-fifth MTP and first IP joint allowed a patient to be classed as having forefoot RA involvement. The same method also applied to midfoot, hindfoot and ankle RA involvement. We also investigated foot deformities, such as hallux valgus, metatarsus primus varus, splayfoot and pes planus. Hallux valgus was defined as a greater than 20° angle of intersection between the longitudinal axis of the first metatarsal and the proximal phalanx. Metatarsus primus varus was defined as a greater than 10° angle of intersection between the first and second metatarsal. Splayfoot was defined as a greater than 35° intermetatarsal angle between the first and fifth metatarsal. Pes planus was defined as less than 20° of calcaneal pitch, which is the angle between the lower surface of the calcaneus and the ground.

### Functional disability as an outcome

Functional disability was evaluated using the Korean version of the Health Assessment Questionnaire-Disability Index (HAQ-DI) [26]. The disability index comprises 20 questions classified into eight categories, including dressing and grooming, arising, eating, walking, hygiene, reach, grip and common daily activities. Each question is rated on a scale ranging from 0 (no difficulty) to 3 (unable to do). The score of HAQ-DI is the mean of the eight category scores.

### Statistical analysis

Inter- and intraobserver agreement was calculated using prevalence-adjusted and bias-adjusted kappa [27]. We compared the clinical features, including HAQ-DI scores, of RA patients with and without the presence of a foot or ankle RA involvement. The chi-square test was used to compare categorical data and the Mann-Whitney U-test was used to compare continuous data between pairs of groups. Then we performed univariable and multivariable logistic regression analyses to find the impact of midfoot and hindfoot RA involvement on functional disability (HAQ-DI ≥ 0.5 as a median) and walking disability (walking score, which is subcategory of HAQ-DI, ≥ 1). Variables significant in the univariable analysis at some arbitrary level (*P* < 0.25) and potential predictors of HAQ-DI (age, disease duration) and foot involvement (BMI, sites of RA involvement) were selected as candidates for the multivariable analysis. All data was analyzed using SPSS for Windows Version 18.0 (SPSS Inc., Chicago, IL, USA) and *P*-values less than 0.05 were considered statistically significant.

## Results

The results for inter- and intraobserver agreement are shown in Additional file 1: Table S1.

### Baseline characteristics and prevalence of foot involvement

A total of 120 patients (113 females, 7 males) with a median age of 48.0 years [interquartile range (IQR), 37–56 years] were included in this study. The median disease duration was 58.0 months (IQR, 10–89 months). Seventy-four patients (61.7%) had a foot or ankle RA involvement in at least one region. Among these, the prevalence of forefoot, midfoot, hindfoot and ankle involvement was 32 (43.2%), 24 (32.4%), 46 (62.2%) and 4 (5.4%), respectively.

The demographic and clinical characteristics of all patients, both with and without foot or ankle involvement, are outlined in Table 1. The group with foot or ankle involvement had higher median visual analogue scale (VAS) scores (30.0 vs. 15.0, *p* < 0.05) and higher median DAS28-ESR scores (4.08 vs. 3.07, *p* < 0.05) than the group without foot or ankle involvement. From the medication histories, only biologic DMARDs were used significantly more often in the foot or ankle involvement group (44.6% vs. 15.2%, *p* < 0.05). Also, health-related quality of life, as assessed by the EQ-5D, was lower in the group with foot or ankle involvement (0.76 vs. 0.81, *p* < 0.05).

**Table 1:** Comparison of baseline characteristics between patients with and without foot or ankle RA involvement

Variables	No RA involvement in the foot or ankle, *n* = 46	RA involvement in the foot or ankle, *n* = 74	*P* value
Age, median (IQR), years	45.5 (36.8–54.0)	50.5 (38.75–58.0)	0.09
Disease duration, median (IQR), months	47.5 (7.50–80.0)	63.0 (12.3–103.3)	0.09
BMI, median (IQR), kg/m^2^	21.7 (20.5–24.0)	22.9 (20.2–25.0)	0.35
Patient global assessment, VAS (0–10), median (IQR)	15.0 (10.0–30.0)	30.0 (10.0–40.0)	<0.05
CRP, median (IQR), mg/dl	0.40 (0.34–0.95)	0.40 (0.15–1.13)	0.97
ESR, median (IQR), mm/h	18.5 (10.0–40.0)	27.0 (11.8–43.0)	0.35
DAS28-ESR, median (IQR)	3.07 (2.50–4.10)	4.08 (3.05–5.07)	<0.05
Female, n (%)	45 (97.8)	68 (91.9)	0.25
RF positive, n (%)	39 (84.8)	65 (87.8)	0.63
ACPA positive, n (%)	42 (91.3)	65 (89.0)	0.86
Medications, n (%)			
Glucocorticoid	35 (76.1)	54 (73.0)	0.71
NSAID	38 (82.6)	67 (90.5)	0.20
Methotrexate	44 (95.7)	67 (90.5)	0.48
Conventional DMARDs^a^	45 (97.8)	72 (97.3)	1.00
Biologic DMARDs^b^	7 (15.2)	33 (44.6)	<0.05
EQ-5D, median (IQR)	0.81 (0.75–0.86)	0.76 (0.69–0.84)	<0.05
HAQ-DI (0–3), median (IQR)	0.38 (0–0.5)	0.63 (0.13–1.16)	<0.05
RA involvement, *n* (%)			
Forefoot	0	32 (43.2)	NA
Midfoot	0	24 (32.4)	NA
Hindfoot	0	46 (62.2)	NA
Ankle	0	4 (5.4)	NA
Foot deformity, *n* (%)			
Hallux valgus,	8 (17.4)	18 (24.3)	0.37
Metatarsus primus varus	16 (34.8)	22 (29.7)	0.56
Splayfoot	0	2 (2.7)	0.52
Pes planus	43 (93.5)	71 (95.9)	0.67

*ACPA* anti-cyclic citrullinated protein antibody, *BMI* body mass index, *CRP* C-reactive protein, *DAS28* disease activity score 28, *DMARDs* disease-modifying antirheumatic drugs, *ESR* erythrocyte sedimentation rate, *EQ-5D* EuroQol five-dimension questionnaire, *HAQ-DI* Health Assessment Questionnaire-Disability Index, *IQR* interquartile range, *NA* not applicable, *NSAID* non-steroidal anti-inflammatory drug, *RF* rheumatoid factor, *SD* standard deviation, *VAS* visual analogue scale

^a^Conventional DMARDs include methotrexate, hydroxychloroquine, sulfasalazine and leflunomide

^b^Biologic DMARDs include adalimumab, etanercept, golimumab, abatacept and rituximab

### Foot involvement and functional disability

**Fig. 1:**
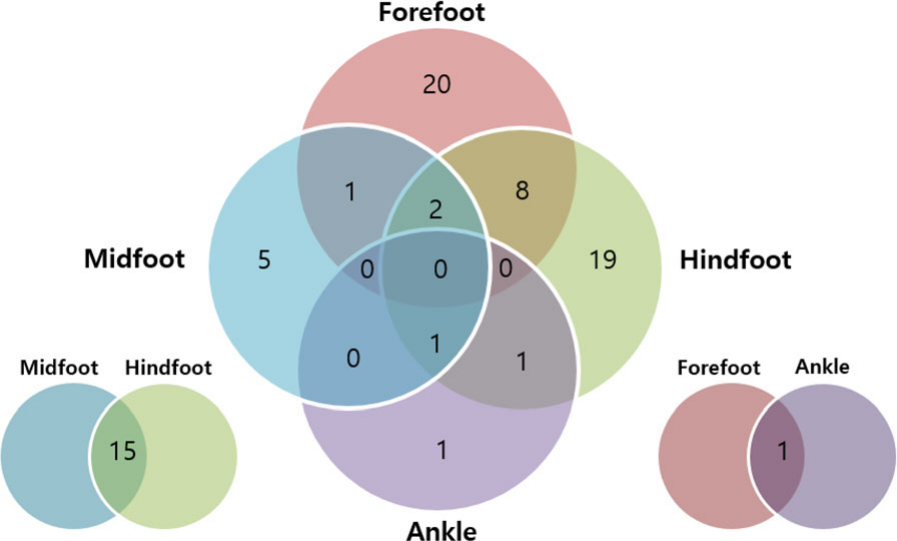
The distribution of RA joint involvement

**Fig. 2:**
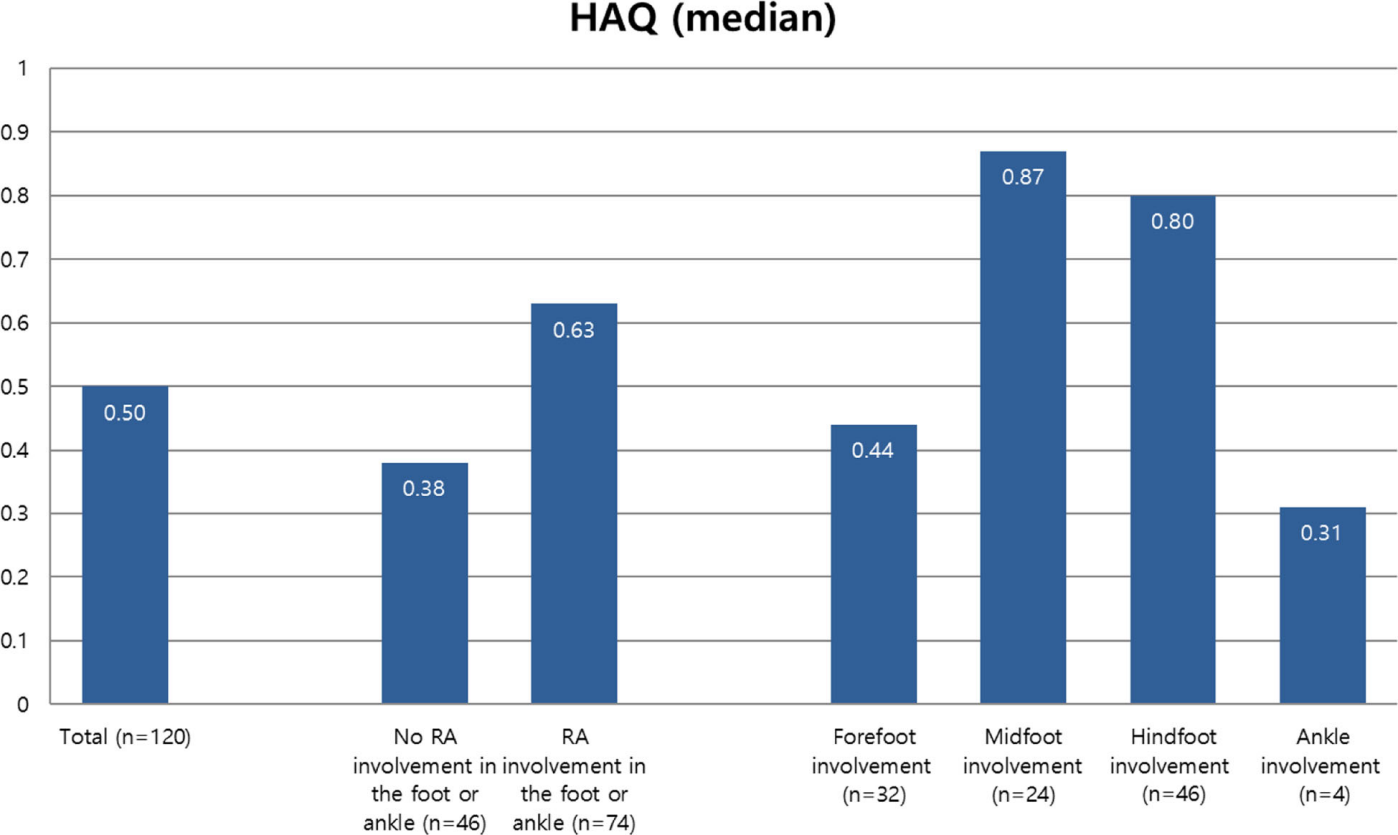
Median Health Assessment Questionnaire (HAQ) scores according to RA involvement in the foot or ankle

The distribution of RA joint involvement is shown in Fig. 1 and median HAQ-DI scores according to the locus of RA involvement are shown in Fig. 2. The median HAQ-DI score was higher in the group with foot or ankle involvement than in the non-involvement group (0.63 vs. 0.38, *p* < 0.05). Patients with midfoot involvement had the highest HAQ-DI score, followed by hindfoot involvement.

### Impact of midfoot involvement and hindfoot involvement on functional disability

In univariable analysis, midfoot involvement corresponded with a 4.68-fold increase and hindfoot involvement corresponded with a 4.66-fold increase in the risk of being in the higher functional disability group (HAQ-DI score ≥ 0.5 as a median) (Table 2). After adjusting for age, disease duration, BMI, RF positivity, glucocorticoid use, biologic DMARD use, and foot deformities, DAS28-ESR [odds ratio (OR) 4.33, 95% confidence interval (CI) 1.69–11.1, *p* < 0.05] and hindfoot involvement (OR 3.14, 95% CI 1.18–8.38, *p* < 0.05) were risk factors for functional disability (Table 2). Additionally, DAS28-ESR (OR 6.88, 95% CI 1.84–25.7, *p* < 0.05), glucocorticoid use (OR 8.24, 95% CI 1.77–38.4, *p* < 0.05) and midfoot involvement (OR 5.81, 95% CI 1.41–23.9, *p* < 0.05) were risk factors affecting walking disability (walking score in HAQ-DI ≥ 1) after adjusting for other clinical variables and foot deformities (Table 3).

**Table 2:** Factors influencing functional disability (total HAQ-DI score ≥ 0.5 as median) in patients with RA (*n* = 120)

Variables	Unadjusted analysis		Adjusted analysis	
	OR (95% CI)	*P* value	OR (95% CI)	*P* value
Age	1.00 (0.97–1.03)	0.87	1.01 (0.97–1.05)	0.78
Disease duration^a^	2.25 (1.02–4.99)	0.05	1.85 (0.59–5.86)	0.29
BMI	1.05 (0.95–1.16)	0.37	1.03 (0.90–1.17)	0.71
DAS28-ESR^b^	6.34 (2.79–14.40)	<0.05	4.33 (1.69–11.10)	<0.05
RF positive	2.67 (0.87–8.22)	0.09	2.34 (0.64–8.64)	0.20
Glucocorticoid	1.70 (0.74–3.88)	0.21	2.09 (0.67–6.58)	0.19
Biologic DMARDs	3.76 (1.65–8.56)	0.00	1.58 (0.51–4.92)	0.43
RA involvement				
Forefoot	0.91 (0.41–2.05)	0.83	1.03 (0.39–2.71)	0.95
Midfoot	4.68 (1.62–13.60)	<0.05	2.02 (0.49–8.29)	0.33
Hindfoot	4.66 (2.07–10.5)	<0.05	3.14 (1.18–8.38)	<0.05
Ankle	0.93 (0.13–6.85)	0.95	0.68 (0.06–7.70)	0.75
Foot deformity				
Hallux valgus	1.67 (0.69–4.06)	0.26	1.03 (0.32–3.33)	0.96
Metatarsus primus varus	1.06 (0.49–2.29)	0.89	1.09 (0.41–2.88)	0.87
Pes planus	2.22 (0.39–12.60)	0.37	1.30 (0.15–10.90)	0.81

*BMI* body mass index, *DAS28* disease activity score 28, *DMARDs* disease-modifying antirheumatic drugs, *ESR* erythrocyte sedimentation rate, *HAQ-DI* Health Assessment Questionnaire-Disability Index, *OR* odds ratio, *RF* rheumatoid factor

^a^Disease duration was divided into two groups based on a cut-off of 2 years

^b^DAS28-ESR was divided into two groups, remission to low disease activity (DAS28-ESR ≤ 3.2) and moderate to high disease activity (DAS28-ESR > 3.2)

**Table 3:** Factors influencing walking disability (walking score of HAQ-DI ≥ 1) in patients with RA (*n* = 120)

Variables	Unadjusted analysis		Adjusted analysis	
	OR (95% CI)	*P* value	OR (95% CI)	*P* value
Age	1.00 (0.97–1.03)	0.99	1.01 (0.97–1.06)	0.56
Disease duration^a^	2.28 (0.93–5.59)	0.07	1.81 (0.48–6.85)	0.39
BMI	1.08 (0.97–1.20)	0.15	1.07 (0.93–1.24)	0.34
DAS28-ESR^b^	10.5 (3.40–32.1)	<0.05	6.88 (1.84–25.7)	<0.05
RF positive	2.39 (0.64–8.94)	0.19	1.82 (0.40–8.37)	0.44
Glucocorticoid	4.59 (1.48–14.2)	<0.05	8.24 (1.77–38.4)	<0.05
Biologic DMARDs	4.21 (1.86–9.51)	<0.05	1.89 (0.54–6.64)	0.32
RA involvement				
Forefoot	0.72 (0.30–1.75)	0.47	0.85 (0.28–2.60)	0.77
Midfoot	6.00 (2.28–15.8)	<0.05	5.81 (1.41–23.9)	<0.05
Hindfoot	2.85 (1.30–6.25)	<0.05	1.24 (0.41–3.76)	0.71
Ankle	0.66 (0.07–6.54)	0.72	0.29 (0.02–5.73)	0.42
Foot deformity				
Hallux valgus	1.33 (0.54–3.28)	0.53	0.68 (0.19–2.51)	0.56
Metatarsus primus varus	0.89 (0.39–2.03)	0.78	0.93 (0.30–2.85)	0.90
Pes planus	1.00 (0.18–5.71)	1.00	0.50 (0.05–5.22)	0.56

*BMI* body mass index, *DAS28* disease activity score 28, *DMARDs* disease-modifying antirheumatic drugs, *ESR* erythrocyte sedimentation rate, *HAQ-DI* Health Assessment Questionnaire-Disability Index, *OR* odds ratio, *RF* rheumatoid factor

^a^Disease duration was divided into two groups based on a cut-off of 2 years

^b^DAS28-ESR was divided into two groups, remission to low disease activity (DAS28-ESR ≤ 3.2) and moderate to high disease activity (DAS28-ESR > 3.2)

## Discussion

The aim of this study was to assess the prevalence of midfoot and hindfoot involvement, and its effect on functional disability in Korean patients with RA. Hindfoot involvement was most common, and midfoot involvement was as common as forefoot involvement. Foot involvement was associated with poor quality of life and functional disability. Of the various groups, those with midfoot involvement had the greatest functional disability, and hindfoot involvement and midfoot involvement were risk factors for functional disability and walking disability, respectively.

We found that a total of 61.7% of patients had foot or ankle RA involvement, which is a somewhat lower prevalence than in previous reports; the reported prevalence rates of RA foot or ankle involvement have varied from 64% to 96.2% [5–7, 28]. The reasons for our lower prevalence are that our patients were relatively young, their disease was of short duration, and many were receiving biological agents. It has been previously reported that the forefoot is most commonly affected in RA [2, 4, 5, 7]. In a previous report, the prevalences of forefoot, midfoot, hindfoot and ankle radiographic involvement in RA were 88%, 62%, 32% and 26%, respectively [4]. However, hindfoot involvement was most common and midfoot RA involvement was as common as forefoot involvement in our study. The difference in the order of frequency of involvement according to location is probably due differences of definition. Since the reported extent and degree of involvement of foot joints varies greatly between studies a consensus definition may be required to permit reliable comparisons between studies, cross-sectional design.

In a previous report, foot and ankle joint destruction was divided into 5 clusters: normal, forefoot, midfoot, mid-hindfoot and combined type. Of these, the mid-hindfoot and combined type deformities showed a significant change in functional disability and decrease in walking ability [6]. Although a slightly different definition of foot involvement was used, the midfoot and hindfoot involvement groups also had greater functional disability in our study. In addition, midfoot involvement and hindfoot involvement were risk factors for walking disability and total functional disability in this study. Therefore, midfoot and hindfoot arthritis is likely to be important for the function and activity levels of RA patients.

A previous study showed that longer disease duration is associated with impaired foot function and reduced walking speed in patients with RA-related foot complaints because of alterations in pressure distribution and gait patterns [29]. Another study reported a correlation between disease duration and hallux rigidus and claw toe, which are typical forefoot deformities [7]. Also, the incidence rate of the pes planus, which is common midfoot deformity, increases with longer disease duration in RA patients [30]. Consistently, our study found that median disease duration was significantly longer in the foot or ankle involvement group than in the non-involvement group.

High BMI was correlated with foot problems in some previous studies [2, 28] because excessive mechanical load has an effect on joint damage. However, we found no significant difference in BMI between RA patients with foot or ankle involvement and without involvement. In addition, BMI was not associated with functional disability.

Over the last decades, treatment for RA has become more aggressive with the advent of biologic DMARDs. One-third of our patients were treated with biologic DMARDs and a higher proportion of patients those with foot or ankle involvement used biologic DMARDs. This result is consistent with other reports [19, 31].

A previous study found that damage in the midfoot occurs suddenly and peaks in cases 5–10 years after RA diagnosis. After that period, midfoot injury rates decrease, while mid-hind foot type injuries gradually increase [6]. In another study, hindfoot involvement was rarely observed in the absence of midtarsal and MTP joint disease [4]. These results support the hypothesis that midfoot involvement may precede hindfoot involvement. Although our results do not support that hypothesis, if it is correct early intervention for patients with midfoot arthritis could prevent hindfoot impairment and rescue functional capacity.

In RA patients with foot arthritis, medical and surgical treatment options are available to decrease pain and improve function. First, it is important to educate patients on how to carefully inspect their feet carefully and about the necessity of need for regular exercise, which is helpful to stretch tendons, strengthen muscles and maintain maximum range of motion [14]. Then, we can provide pain relief and resolve joint inflammation through pharmacologic treatments. Orthotic devices, such as shoes, canes and crutches, are useful in ambulation and can provide support to the arch, accommodate a deformed foot and relieve pain. Surgical treatment, including synovectomy and reconstruction, are also helpful in cases of severe inflammation and progressive joint destruction [8, 12, 13, 32]. Our study highlights the importance of focusing on midfoot and hindfoot problems, and not exclusively forefoot problems, in routine clinical care with physical and radiographic examination for early detection and treatment.

There are a few limitations to our study. First, it had a cross-sectional design and involved a relatively small number of patients. Second, we could not evaluate additional radiographic views such as foot oblique and ankle AP view for more accurate assessment. Third, we could not use quantitative radiographic scoring methods such as the Sharp/van der Heijde Score or Larsen score. Because these scoring system focus on forefoot deformity rather than midfoot or hindfoot deformity. And we lacked information about current treatments for foot arthritis in terms of the use of orthoses, footwear and steroid injection. However, because more than half of the patients had some degree of foot involvement, we were able to use a multivariable model to identify whether foot involvement was a risk factor for functional impairment. Our study is also meaningful in that it is the first study to assess the impact of foot involvement in the three foot sub-sections on functional disability in Korean RA patients. The present study highlights the importance for early detection of focusing on midfoot and hindfoot problems in routine clinical care, together with physical and radiographic examination, rather than exclusively on forefoot problems. Early detection of foot involvement can reduce disability through early treatment. This study may guide a future longitudinal study designed to evaluate how progression of foot involvement impacts on the outcomes of RA patients using radiographic assessment tools that distinguish between RA and osteoarthritis foot involvement.

## Conclusions

Although the foot is not examined in the clinical assessment of disease activity in RA, foot abnormalities are seen frequently and may affect disability in RA. In particular, hindfoot involvement is associated with functional disability and midfoot involvement affects walking. Foot involvement remains a great problem even though a large number of patient with RA are now being treated with biological agents. We suggest that foot involvement should be regularly monitored in order to reduce the functional disability of RA patients.

## Additional file

Additional file 1: Table S1. Interobserver and intraobserver agreement for assessment of radiographs. (DOCX 16 kb)

## Abbreviations

ACPA: Anti-cyclic citrullinated peptide antibody
ACR: American College of Rheumatology
BMI: Body mass index
CI: Confidence interval
CRP: C-reactive protein
DAS: Disease activity score
DMARDs: Disease-modifying antirheumatic drugs
EQ-5D: EuroQol five-dimension questionnaire
ESR: Erythrocyte sedimentation rate
EULAR: European League Against Rheumatism
HAQ-DI: Health Assessment Questionnaire-Disability Index
IP: Interphalangeal
IQR: Interquartile range
MTP: Metatarsophalangeal
OR: Odds ratio
PABAK: prevalence-adjusted and bias-adjusted Kappa
RA: Rheumatoid arthritis
RF: Rheumatoid factor
TMT: Tarsometatarsal
VAS: Visual analogue scale
