# Supplementary material for: Impact of midfoot and Hindfoot involvement on functional disability in Korean patients with rheumatoid arthritis
Source: BMC Musculoskelet Disord. 2017 Aug 24;18:365. doi: 10.1186/s12891-017-1726-7 (PMC5571626; doi:10.1186/s12891-017-1726-7)
Supplement: Additional file 1: Table S1. — Interobserver and intraobserver agreement for assessment of radiographs. (DOCX 16 kb) [file 12891_2017_1726_MOESM1_ESM.docx]

Additional file 1: Table S1. Interobserver and intraobserver agreement for assessment of radiographs.

| Joint | Observer 1× Observer 1 | | Observer 2 × Observer 2 | | Observer 1 × Observer 2 | |
| --- | --- | --- | --- | --- | --- | --- |
|  | PABAK | % Agreement | PABAK | % Agreement | PABAK | % Agreement |
| 1st IP | 0.81 | 83.3 | 0.87 | 93.3 | 0.60 | 83.3 |
| 1st MTP | 0.53 | 76.7 | 0.93 | 96.7 | 0.33 | 66.7 |
| 2nd MTP | 0.80 | 90.0 | 0.80 | 90.0 | 0.80 | 90.0 |
| 3rd MTP | 0.87 | 93.3 | 0.73 | 86.7 | 0.73 | 86.7 |
| 4th MTP | 0.87 | 93.3 | 0.87 | 93.3 | 0.80 | 90.0 |
| 5th MTP | 0.73 | 86.7 | 0.87 | 93.3 | 0.60 | 80.0 |
| 1st TMT | 0.93 | 96.7 | 1.00 | 100 | 0.83 | 91.7 |
| 2nd TMT | 0.87 | 93.3 | 1.00 | 100 | 0.83 | 91.7 |
| 3rd TMT | 1.00 | 100 | 1.00 | 100 | 0.77 | 88.3 |
| 4th TMT | 0.87 | 93.3 | 0.93 | 96.7 | 0.83 | 91.7 |
| 5th TMT | 0.87 | 93.3 | 0.93 | 96.7 | 0.78 | 89.2 |
| Naviculocuneiform | 1.00 | 100 | 0.87 | 90.0 | 0.87 | 93.3% |
| Talonavicular | 0.93 | 96.7 | 1.00 | 100 | 0.93 | 96.7 |
| Calcaneocuboidal | 1.00 | 100 | 0.87 | 93.3 | 1.00 | 100 |
| Talocalcaneal | 0.73 | 86.7 | 0.87 | 93.3 | 0.67 | 76.7 |
| Tibiotalar | 0.87 | 93.3 | 1.00 | 100 | 0.80 | 90.0 |

*IP* interphalangeal, *MTP* metatarsophalangeal, *PABAK* prevalence-adjusted and bias-adjusted kappa, *TMT* tarsometatarsal, *% agreement* percent agreement
